# The Strengths and Barriers Recovery Scale (SABRS): Relationships Matter in Building Strengths and Overcoming Barriers

**DOI:** 10.3389/fpsyg.2021.663447

**Published:** 2021-03-26

**Authors:** David Best, Arun Sondhi, Lorna Brown, Mulka Nisic, Gera E. Nagelhout, Thomas Martinelli, Dike van de Mheen, Wouter Vanderplasschen

**Affiliations:** ^1^Department of Criminology, College of Business, Law and Social Sciences, University of Derby, Derby, United Kingdom; ^2^Therapeutic Solutions (Addictions), London, United Kingdom; ^3^Recovered Users Network (RUN), Brussels, Belgium; ^4^IVO Research Institute, The Hague, Netherlands; ^5^Department of Health Promotion, Maastricht University (CAPHRI), Maastricht, Netherlands; ^6^Tranzo, Tilburg University, Tilburg, Netherlands; ^7^Recovery and Addiction Cluster, Department of Special Needs Education, Ghent University, Ghent, Belgium

**Keywords:** addiction, recovery, connectedness, social relations, strengths, substance use disorder, barriers

## Abstract

There is a well-established relationship between isolation and both morbidity and mortality in the context of addiction recovery, yet the protective effects of intimate and familial relationships have not been adequately assessed. The current paper uses the European Life In Recovery database to assess the association between relationship status and living with dependent children on recovery capital of people in recovery from drug addiction, operationalised by the Strengths And Barriers Recovery Scale (SABRS). The study participants were drawn from the REC-PATH study and supplemented by a second sample recruited by the Recovered Users Network (RUN) across various European countries, resulting in a combined sample of 1,313 individuals completing the survey, primarily online. The results show that, in recovery, those who are married or co-habiting reported significantly greater recovery strengths and fewer barriers to recovery, and reported greater gains in recovery capital across their recovery journeys. Similar associations are found for participants who have dependent children living with them. There is also some indication that this association is stronger for female than for male participants. Finally, having more people that one can rely on and a greater proportion of people in recovery in the social network are both linked to greater recovery capital and greater self-reported growth in recovery capital. We conclude that this study provides further evidence in favour of a “social cure” in recovery, in which close familial ties are associated with stronger recovery resources.

## Introduction

Recovery from drug addiction is an emerging area of empirical research. There is a growing consensus on definitions, with general agreement that recovery is a journey characterised by dynamic and non-linear growth in health and wellbeing, sobriety and active participation in a range of social and societal roles and activities (Betty Ford Institute Consensus Group, 2007; UK Drug Policy Commission, 2008; Sheedy and Whitter, 2009; Ashford et al., 2019; Dekkers et al., 2020). The Betty Ford Institute Consensus Group (2007) went further by categorising recovery by duration into “early recovery” (<1 year), “sustained recovery” (1–5 years) and “stable recovery” (>5 years), with the implication that recovery is more robust as the individual progresses through these stages. A number of evidence-based components of the recovery process have been identified. A review by Humphreys and Lembke (2014) indicates the importance of peer and social processes, with the authors showing evidence for peer-based interventions, mutual aid and recovery housing. Another approach to recovery-focussed interventions can be drawn from the mental health field (Leamy et al., 2011; Best, 2019) and is based on the acronym CHIME (Connections, Hope, Identity, Meaning, Empowerment), in which human connection (normally from a peer or peer group) generates a sense of hope that recovery is achievable and—in turn—motivates a virtuous circle of meaningful activities, an emerging sense of empowerment and a positive identity.

The emergence of recovery science has also prompted an interest in metrics, one of which is the concept of “recovery capital.” Granfield and Cloud (2001) first used this term in 2001 in discussing the concept of natural recovery and subsequently defined recovery capital as “the sum total of one’s resources that can be brought to bear on the initiation and maintenance of substance misuse cessation” (Cloud and Granfield, 2008, p. 1972). The notion that this term could be quantified was first mooted by Best and Laudet (2010), who suggested that there were three domains for recovery capital—personal, social and community capital—but that these were dynamically related and included positive as well as negative elements. Groshkova et al. (2012) published the psychometric properties of the Assessment of Recovery Capital (ARC), an instrument designed to measure both strengths and barriers (across ten domains of personal and social recovery capital) and to measure progress in the recovery journey, and demonstrated that this was a robust and reliable tool. More recently, the ARC tool has been embedded in the REC-CAP (Recovery Capital) tool, an online instrument that not only assesses recovery capital but that is embedded in a care planning model that encourages clinicians and peer mentors to plan and support the ongoing accrual of positive recovery capital (Cano et al., 2017). Cano and colleagues also demonstrated the critical role that engagement in meaningful activities can play in the building of strengths and the resulting increases in wellbeing. In her review of the existing literature on recovery capital, Hennessy (2017) concluded that “as a framework for describing the various resources and supports that can be accumulated or exhausted to support recovery, RC [Recovery Capital] provides a broad overview of the multiple, interrelated factors in the recovery process and could be used as a tool to untangle variegated recovery pathways” (2017, p. 358).

To further complement the existing measures and tools, the current paper builds on innovative work described by Best et al. (2020) on the use of the Strengths And Barriers Recovery Scale. The SABRS scale is based on the Life in Recovery (LiR) survey method, first reported by Laudet (2013) in the United States, which assesses experiences in five life domains (work, finances, legal status, family and social relations, and citizenship), recording respondents’ perceptions both retrospectively for their time in active addiction and currently when they are in recovery. The original LiR survey has been used in a number of other countries (e.g., United Kingdom, Best et al., 2015a; Canada, McQuaid et al., 2017; Australia, Elms et al., 2018), and most recently as part of a European study of recovery pathways (REC-PATH, Best et al., 2018). In the REC-PATH study (Recovery Pathways and social responses in the United Kingdom, the Netherlands, and Belgium), the Life in Recovery survey was used as a recruitment and screening tool for studying recovery from problem drug use (Martinelli et al., 2020a). The survey was also deployed by the Recovered Users Network (RUN) across a number of other European countries to assess recovery experiences and wellbeing. Using the latter dataset, the SABRS scale was created by dividing relevant items into strengths and deficits questions and generating change measures by subtracting the active addiction scores from recovery measures (e.g., change in involvement in family activities) (Best et al., 2020). All items that had a positive valence (such as “I exercise regularly”) were categorised as Recovery Strengths and all items that had a negative valence (such as “I have been to prison”) were categorised as Recovery Barriers. There were no neutral items. As each item was simply endorsed or not, this allowed a simple tally of Recovery Strengths and Recovery Barriers at two time points—“In active addiction” and “In recovery.” A proxy measure of change could then be calculated by subtracting each “In active addiction” composite score from each “In recovery score,” generating overall change scores for Recovery Strengths and Recovery Barriers.

Although there is a clear relationship between social connectedness and wellbeing in the general population (Christakis and Fowler, 2009; Holt-Lunstad et al., 2011; Jetten et al., 2012), there is limited research on this association in addiction and recovery populations. Available research shows that the extent to which the individual exhibits a sense of group belonging with peers in therapeutic communities (referred to as social identification) is predictive of positive outcomes (Dingle et al., 2014; Beckwith et al., 2015). Similar findings have been reported for mutual aid group participation (Kelly, 2019; Martinelli et al., 2020b; Barrett and Murphy, 2021). In their study of mental health recovery among people labelled as not criminally responsible, Aga et al. (2021) found that connectedness is central to the recovery experience, including a sense of belonging that is linked to active engagement in social groups and society at large. Taking care of children has been identified as a major barrier to seeking treatment for women (because of concerns of involvement of social services and the perceived threat of child removal), as well as an important factor promoting treatment retention and recovery in mother-child programs (Neale et al., 2018; Andersson et al., 2020; Schamp et al., 2020), where reunification with children or retaining custody of children can be a strong motivation to strive for recovery.

To assess the role of human connection in addiction recovery, we have combined the data from the RUN dataset with the screening data from the REC-PATH study discussed above to examine the associations between recovery capital measured using the SABRS tool and a number of indicators of social support. The research questions to be addressed in this paper are:

RQ1: To what extent do recovery strengths and barriers change in recovery and is this a function of recovery duration?

RQ2: Do people in recovery who are in a relationship differ in recovery strengths and barriers from people who are not, and is this associated with the extent of change in both recovery strengths and recovery barriers in the period between active addiction and recovery?

RQ3: Do people in recovery who live with their dependent children differ in recovery strengths and recovery barriers from people who do not live with children, and is this associated with the extent of change in both recovery strengths and recovery barriers in the period between active addiction and recovery?

RQ4: Do people in recovery with strong social support networks differ in recovery strengths and recovery barriers from people with weaker support networks, and is this associated with the extent of change in both recovery strengths and recovery barriers in the period between active addiction and recovery?

RQ5: What types of social networks and supports are closely related to positive recovery capital?

RQ6: What social factors are linked with growth in recovery strengths?

## Materials and Methods

### Design and Procedure

The paper is based on a convenience sample initially recruited during the REC-PATH study, an EU-funded multi-country and multi-method study on recovery pathways and experiences among persons with a history of illicit drug addiction. Between January and June 2018, the Life In Recovery (LiR) survey was used as a recruitment and screening instrument) in the United Kingdom, the Netherlands, and Flanders (Dutch-speaking part of Belgium) (*n* = 776). It was also distributed through the international Recovered Users Network (RUN), after it was translated into a number of other European languages (Bosnian/Croatian/Serbian/Montenegrin, Swedish, Polish, Portuguese, and Spanish, besides English and Dutch). RUN is a civil society organisation that promotes recovery among individuals, agencies and organisations, primarily but not exclusively in Eastern Europe. Five hundred and thirty seven individuals were recruited through the RUN network, primarily in Serbia (*n* = 123), Poland (*n* = 79), Bosnia (*n* = 72), and Spain (*n* = 60). The total sample for this study consisted of 1,313 participants.

The survey was available online on the REC-PATH project website^1^, as well as through hard copies. Study participation was promoted in various ways through recovery groups and organisations, drug services, social media, websites, TV shows and other partner agencies. Snowball sampling was used to reach out to a more diverse group of potential participants. We used the online platform Qualtrics for data collection. Participants could choose which language they wanted to complete the form in, upon accessing the project website. Online information and consent preceded initiation of the survey. For participants to complete the form, each item of each section required an endorsement or they would not be able to pass onto the next question. Consequently, only completed questionnaires were available on the online platform. Hard copies of the survey were made available for those who did not have access to or were not comfortable completing the online survey. Only completed hard copies were entered into the database. Thus, no missing data had to be managed in the analysis. Data are based on self-reported survey completion and no financial incentive was provided for study participation. More information on the procedure for the REC-PATH (Best et al., 2018; Martinelli et al., 2020a) and RUN data collection (Best et al., 2020) can be found elsewhere.

### Instrument

As outlined in the original SABRS paper (Best et al., 2020), the 44 items in the Life in Recovery survey were reduced to 32 items, consisting of 15 strengths items and 17 deficit items (with all items either endorsed or not), creating a scale of 0–15 for strengths and 0–17 for deficits (see Table 1). The retrospective approach of the Life in Recovery method looks at these strengths and deficits both during active addiction and in recovery, meaning that there are four scores derived from the scale:

**TABLE 1:** Final set of included items (*n* = 32) in the Strengths And Barriers Recovery Scale (SABRS).

Recovery Strength items	Recovery Barrier items
– Exercise regularly – Have a GP – Have regular dental checks – Have good nutrition – Take care of your health – Maintain a driving licence – Maintain a bank account – Able to pay your bills – Maintain stable housing – Remain in steady employment – Further your education or training – Start your own business – Participate in family life – Plan for the future – Volunteer	– Have untreated emotional or mental health problems – Make regular visits to the emergency room – Regular use of health services – Smoke – Have your drivers’ licence revoked – Drive under the influence of alcohol or drugs – Damage property – Been arrested – Been charged with a criminal offence – Been to prison – Have bad debts – Were unable to pay the bills – Regularly missed school or work – Dropped out of school or college – Fired or suspended from work – Lose custody of children – Experience family violence

1. Recovery Strengths in Active Addiction.
2. Recovery Deficits in Active Addiction.
3. Recovery Strengths in Recovery.
4. Recovery Deficits in Recovery.

The four domain scores allow a change analysis to be conducted, where the growth in strengths can be calculated as the total of Recovery Strengths in Recovery minus the total of Recovery Strengths in Active Addiction. Similarly, the change in Recovery Deficits is calculated as the total of Recovery Deficits in Recovery minus the total Recovery Deficits in Active Addiction.

### Data-Analysis

The current analysis consists of three components. First, we provide a socio-demographic description of the people completing the survey, and the social networks and supports associated with people in recovery. The sample was divided into three groups: those in early (<1 year), sustained (1–5 years) and stable recovery (>5 years) (Betty Ford Institute Consensus Group, 2007). Second, analyses of variance assess differences associated with changes in recovery strengths and barriers (RQ 1–5). Third, we performed a multi-variate analysis to assess predictors of overall growth in recovery strengths to address research question 6. Given the importance of recovery strengths as a prognostic factor, a linear regression model (Table 6) delved further into other variables from the LiR that may be associated with growth in recovery strengths. “Growth in recovery strengths” was calculated as the difference between recovery strengths and addiction strengths. Variables were declared “statistically significant,” if its *p* < 0.05 (i.e., working at 5% significance level). A linear regression model describes in detail all factors associated with growth (increase) in recovery strengths. The variables included in the regression analysis were demographic factors (age, gender, education); country of residence (grouped into the Netherlands and Belgium, Balkans, United Kingdom, Spain and Portugal, and Poland); relationship factors (parenting status, relationship status); addiction career events (age of first and last use of illicit drugs, length of recovery, duration of drug using career); recovery mediators (housing, criminal justice involvement, injecting, education and employment) and types of treatment received (12-step, out-patient, peer support and combinations of interventions).

## Results

### Sample Characteristics

A total of 1,313 participants (combined over the two studies) completed the Life in Recovery survey—consisting of 854 men (65.0%), 453 women (34.5%), and 6 individuals (0.5%) who identified as another gender. The mean age of the sample was 40.3 years (±10.49), with a range of 18–74 years. The REC-PATH sample was drawn from the Netherlands (*n* = 231, 17.6%), Belgium (*n* = 181, 13.8%), and the United Kingdom (*n* = 364, 27.8%). The RUN international sample came from Serbia (*n* = 123, 9.4%), Poland (*n* = 79, 6.0%), Bosnia and Herzegovina (*n* = 72, 5.5%), Spain (*n* = 60, 4.6%), Croatia (*n* = 53, 4.0%), Sweden (*n* = 44, 3.4%), Montenegro (*n* = 15, 1.1%), Portugal (*n* = 6, 0.5%) and also included 85 persons (6.5%) from other European countries.

In terms of relationship status, the largest group were single and never married (*n* = 537, 40.9%) while 300 people (22.8%) were married, 213 (16.2%) co-habiting, 198 (15.0%) divorced or separated, 17 (1.3%) widowed and 48 (3.7%) in other relationship situations. For the purpose of the current analysis, these categories were summarised into 40.9% single, 39.8% married or co-habiting, 16.4% widowed, divorced or separated and 3.0% in another category.

Participants were asked three further questions about their level and type of social contact, with 70 respondents (5.3%) reporting that they had nobody to discuss important things with, 58 (4.4%) reporting that they had one person to discuss important things with, 131 (10.0%) two people, 142 (10.8%) three people and 912 (69.5%) reporting that they had four or more people they could discuss important things with.

The second aspect of social networks that was assessed asked how many of the people the respondent spent time with were users of illicit drugs. The largest group reported that none of the people in their network used illicit drugs (*n* = 779, 59.3%), with 369 (28.1%) reporting that it was less than half, 60 (4.6%) that it was about half, 49 (3.7%) that it was more than half and 56 (4.3%) that it was all of the people they spent time with. The final measure of social connection was an item assessing the proportion of the social network that included people in recovery. For 191 individuals (14.5%), this was “all” of the social network, for 439 participants (33.4%) it was more than half, for 165 (12.6%) it was around half, for 292 (22.2%) it was less than half and for 226 (17.2%) none of the people they spent time with were in recovery.

Table 2 provides the basic summary scores for strengths and barriers both at the time of active addiction and at the time of completing the survey when in recovery.

**TABLE 2:** Number of strengths and barriers while in addiction and recovery (*n* = 1,313).

	Strengths (addiction)	Strengths (recovery)	Barriers (addiction)	Barriers (recovery)
Mean	4.71	10.53	8.59	2.58
*SD*	2.91	3.25	3.30	2.31
Minimum	0	0	0	0
Maximum	15	15	17	17

### Recovery Strengths and Barriers in Active Addiction and in Recovery

Overall, participants reported a mean “increase” of 5.81 strengths (±3.11) and a mean “reduction” of 6.02 barriers (±3.87) between their period in active addiction and recovery. There was an inverse correlation of −0.55 (*p* < 0.001) between changes in strengths and changes in barriers. In other words, the greater the growth in recovery strengths, the greater the reductions in recovery barriers. However, the picture is not consistent across the whole sample and as anticipated, the greater the duration (stability) of recovery the more strengths have accrued (see Table 3).

**TABLE 3:** Mean number of strengths and barriers while in recovery and growth of strengths and reduction of barriers, by recovery stage (*n* = 1,313).

	Early recovery	Sustained recovery	Stable recovery	*F*, significance
Strengths	8.59	10.46	11.69	102.39, *p* < 0.001
Barriers	3.07	2.58	2.33	11.19, *p* < 0.001
Change in strengths	3.33	5.66	7.37	109.84, *p* < 0.001
Changes in barriers	–4.74	–6.13	–6.64	24.50, *p* < 0.001

*Post hoc* testing with Scheffe tests revealed that, for strengths, there were significant differences between each pairwise comparison, but for barriers, there were only significant differences between the early recovery group and the sustained and stable groups. No significant differences were observed between the sustained and stable groups in terms of their barriers to recovery. *Post hoc* tests revealed that all sub-group comparisons were significantly different for strengths change, but for changes in barriers, the significant differences were found between the early and stable group and between the early and sustained group, but not between the stable and sustained groups.

### Relationship and Parenting Status and Recovery Strengths and Barriers

A further analysis assessed the association between relationship status and strengths and barriers, both in active addiction and in recovery, with the results shown in Table 4.

**TABLE 4:** Mean number of strengths and barriers while in recovery and changes in strengths and barriers from addiction to recovery, by relationship status (*n* = 1,313).

	Single	Married or cohabiting	Separated, divorced or widowed	Other	*F*, significance
Strengths in recovery	9.8	11.5	10.6	10.7	31.37, *p* < 0.001
Barriers in recovery	2.8	2.3	2.8	2.4	3.66, *p* < 0.05
Change in strengths	5.0	6.9	5.4	5.4	20.00, *p* < 0.001
Changes in barriers	–5.7	–6.4	–5.8	–6.4	3.64, *p* < 0.05
					

We found a clear association between being in a stable relationship (married or cohabiting) and both higher levels of recovery strengths and lower numbers of residual barriers in recovery. In addition, compared with persons who were not in a stable relationship, these individuals show greater change in strengths and deficits on the journey from addiction to recovery.

From the overall sample, 452 participants (35.6%) reported that they had dependent children living with them (with a mean of 1.73 dependent children living with participants who did have dependent children). Differences by parenting status are shown in Table 5.

**TABLE 5:** Mean number of strengths and barriers while in recovery and changes in strengths and barriers from addiction to recovery, by parenting status (*n* = 1,313).

	No dependent children (mean, SD)	With dependent children	*T*, significance
Strengths in recovery	10.2 (3.32)	11.1 (3.05)	5.03, *p* < 0.001
Barriers in recovery	2.62 (2.06)	2.51 (2.40)	0.84, 0.40
Change in strengths	5.43 (4.00)	6.51 (4.23)	4.61, *p* < 0.001
Change in barriers	−5.79(3.85)	−6.42(3.88)	2.84, *p* < 0.01
			

**TABLE 6:** Linear regression model of growth in recovery strengths.

Prognostic variables	Coefficient	Standard error	P > [t]
Age first using a substance	0	0.001	0.799
Age last using a substance	0.002	0.001	0.139
Duration of substance use (years)	–0.001	0.001	0.043
Length of recovery (years)	0.058	0.01	< 0.0001
Age	0.001	0.001	0.281
Addiction strengths	0.231	0.025	< 0.0001
Addiction deficits	0.275	0.023	< 0.0001
Recovery deficits	–0.398	0.031	< 0.0001
Male	–0.691	0.143	< 0.0001
Secondary education	0.926	0.474	0.051
Higher education	1.218	0.479	0.011
Primary education	0.514	0.492	0.296
Single	–0.044	0.264	0.868
Co-habitation	0.424	0.29	0.145
Married	0.651	0.284	0.022
Divorced	–0.077	0.302	0.798
Living with dependent child	0.192	0.151	0.205
Acute housing need (in last 30 days)	–1.239	0.369	0.001
Has been evicted (in last 30 days)	–0.698	0.457	0.127
Injected (in last 30 days)	–0.846	0.6	0.159
Offended (in last 30 days)	–0.437	0.339	0.197
Criminal justice involvement (in last 30 days)	–0.804	0.294	0.006
Full-time employment (in last 30 days)	1.097	0.149	< 0.0001
Part time employment (in last 30 days)	0.49	0.172	0.005
Undertook education (in last 30 days)	0.87	0.162	< 0.0001
Volunteered (in last 30 days)	0.422	0.146	0.004
Residence: United Kingdom	0.643	0.253	0.011
Residence: Balkans	–0.967	0.271	< 0.0001
Residence: The Netherlands and Belgium	0.554	0.25	0.027
Residence: Spain and Portugal	0.454	0.395	0.25
Residence: Poland	–0.753	0.346	0.03
Received 12-Step help/treatment	0.616	0.373	0.099
Received out-patient (OP) help/treatment	–0.312	0.285	0.273
Received OP and Residential Rehab (RR) help/treatment	0.035	0.215	0.871
Received OP and RR help/treatment	0.063	0.22	0.775
Received RR, OP, 12-step and peer support	0.653	0.237	0.006
Constant	5.942	0.594	0

Participants living with dependent children reported significantly more strengths in recovery than those without dependent children and also showed greater growth in strengths and larger reductions in barriers from active addiction to recovery. However, a significant difference in the number of barriers while in recovery was not found between the two groups.

This analysis was repeated separately for men and women. While 276 men (32.3%) lived with dependent children, relatively more women (*n* = 195; 43.0%) were in this situation, a statistically significant difference (chi^2^ = 15.77, *p* < 0.001). For men, the same overall pattern applied with men living with dependent children reporting more strengths in recovery (11.1 vs. 9.8; *t* = 5.20, *p* < 0.001) and showed a greater increase in strengths from addiction to recovery (6.2 vs. 5.1; *t* = 3.64, *p* < 0.001), and a greater reduction in recovery barriers from active addiction to recovery (−6.7 vs. −6.0; 5 = 2.53, *p* < 0.05). No significant difference in the number of barriers in recovery was experienced by men, while a significant difference was found among women with a greater increase in strengths for women with dependent children than for those without (7.0 vs. 6.3; *t* = 2.00, *p* < 0.05).

### Current Social Networks and Support and Changes in Recovery Strengths and Barriers

**FIGURE 1:**
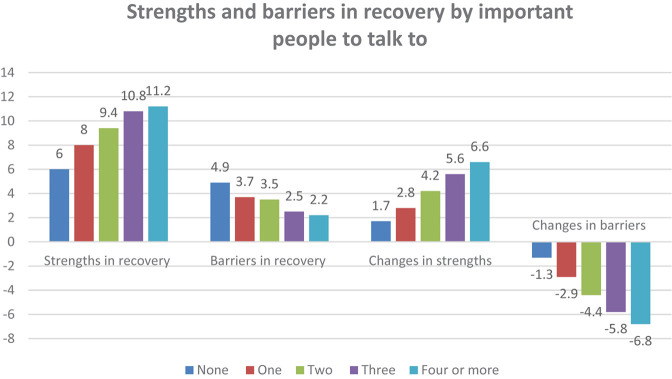
Strengths and barriers in recovery by important people to talk to.

**FIGURE 2:**
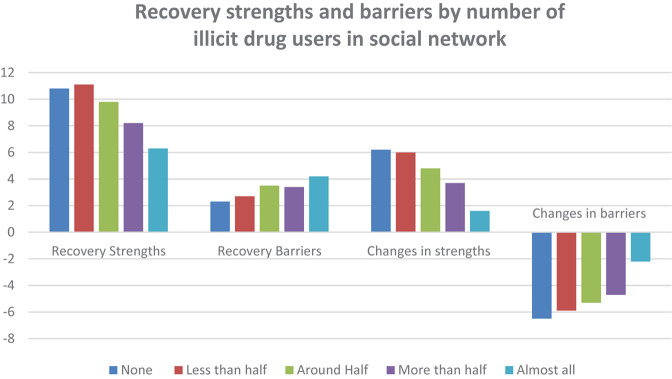
Recovery strengths and barriers by number of illicit drug users in social network.

**FIGURE 3:**
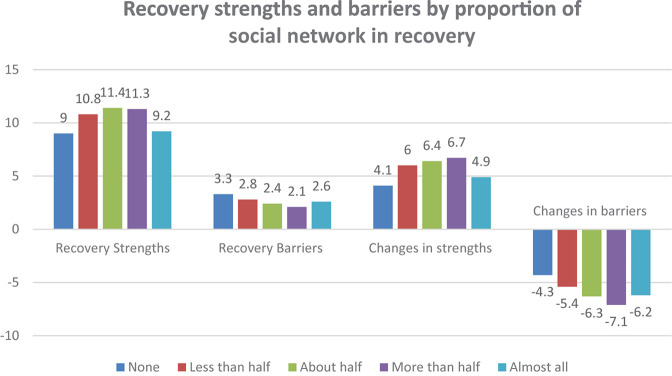
Recovery strengths and barriers by proportion of social network in recovery.

All three measures of current social networks and social support are strongly related to the four SABRS domain scores as shown in Figures 1–3.

Having more people to talk to about important things was strongly associated with greater strengths in recovery (*F* = 66.87, *p* < 0.001), fewer barriers to recovery (*F* = 36.36), more growth in strengths from active addiction to recovery (*F* = 43.44, *p* < 0.001) and greater reductions in barriers to recovery (*F* = 62.17, *p* < 0.001). Figure 2 shows a similar relationship for the number of current users the participant is in contact with while in recovery.

Where respondents have fewer of the current social network members that are active drug users, there was a strong association with greater strengths in recovery (*F* = 38.91, *p* < 0.001), fewer barriers to recovery (*F* = 15.47, *p* < 0.001), more growth in strengths from active addiction to recovery (*F* = 22.54, *p* < 0.001) and greater reductions in barriers to recovery (*F* = 19.30, *p* < 0.001). Figure 3 shows similar associations for the number of contacts with people in recovery while in recovery.

Having fewer members of the current social network who are active drug users was strongly associated with greater strengths in recovery (*F* = 35.96, *p* < 0.001), fewer barriers to recovery (*F* = 9.99, *p* < 0.001), more growth in strengths from active addiction to recovery (*F* = 19.05, *p* < 0.001) and greater reductions in barriers to recovery (*F* = 24.43, *p* < 0.001). However, this only seems to be a linear effect up to the point of having a majority of your friends in recovery. For people who have all of their friends in recovery, the benefits are not as strong.

### Factors Associated With Growth in Strengths

Based on the linear regression analysis, variables that were positively associated with increased “growth” in recovery strengths were shorter duration of substance misuse (in years) and more time in recovery; the baseline level of strengths (higher) and deficits (lower); being female; being married; higher levels of education and either part-time or full-time work; or being in education or volunteering; living in the United Kingdom, the Netherlands, or Belgium; and having participated in residential rehabilitation, peer-based mutual aid, out-patient treatment and peer support groups (see Table 6). In contrast, variables that were negatively associated with “growth” (i.e., lower growth rates of recovery strengths from addiction to recovery) were longer duration of substance misuse in years; more deficits in recovery; acute housing needs; involvement in the criminal justice system and, living in the Balkan countries or Poland.

## Discussion

Few studies have explored the association between social and family relationships and recovery strengths and barriers. The data presented in this paper use the SABRS measure (Best et al., 2020) to demonstrate clear changes in recovery strengths and barriers from active addiction to recovery as an indicator of positive and negative recovery capital (Best and Laudet, 2010). The key findings from this large European sample show that being in two different kinds of close relationships (having an intimate partner and having children) is associated with greater positive changes in recovery strengths and greater reductions in barriers to recovery. Similarly, larger social networks of people in recovery and more people to confide in (indicators of social capital) are associated with more positive growth in recovery strengths and reductions in barriers to recovery.

These findings are consistent with a previous publication on the SABRS scale (Best et al., 2020) indicating—on a much larger sample—that the transition from addiction to recovery is associated both with an increase in recovery strengths and a reduction in recovery deficits. However, and one of the key purposes of measuring recovery capital is that, these transitions are not consistent across the entire sample in predictable ways. While the previous paper primarily focussed on gender effects, the current paper shows clear associations with key social and family factors, consistent with existing evidence about the importance of social support and group belonging in other substance using populations (Jetten et al., 2012; Best et al., 2015b).

There seems to be a beneficial effect for family connections both in terms of relationship status and living with dependent children, suggesting the potential benefits of specifically family connections but also more generally of positive and pro-social relationships for the development and emergence of recovery capital consistent with a “social cure” model (Jetten et al., 2012) and with the application of this model to addiction recovery populations (Dingle et al., 2014; Beckwith et al., 2015). The effects of both relationships and family fit with a model of “informal social control” (Sampson and Laub, 2003), in which positive relationships to family bind people into prosocial lifestyles and support efforts at rehabilitation and reintegration. However, as the study applied a cross-sectional design, causal inference is not warranted. Alternatively, an inverse causal relation may exist, i.e., people with more recovery strengths may be more able to build and maintain social relationships.

Nonetheless, these findings are consistent with work done in the area of desistance from offending by Sampson and Laub (2003) around the importance of “informal social control,” and the multi-variate analysis suggests benefits of marriage on recovery strengths while in recovery. What this model suggests is that family supports create both a pressure on former offenders to conform, but also reduce the opportunities for engaging with former using and offending friendship groups. Yet, LeBel et al. (2008) have cautioned against placing too much emphasis on marriage or parenting as the “causes” of desistance or recovery, suggesting that these key events can be markers of changes that have taken place rather than causes of subsequent change. In the context of recovery capital, it is important to recognise the limitation about whether parenting or relationships have started since the onset of recovery and so we can make no assumptions about causal ordering of this association.

Where we found stronger evidence, and evidence that is consistent with existing empirical and conceptual work (Best et al., 2008, Best et al., 2015b, Longabaugh et al., 2010), is around the importance of moving away from social networks involved in substance use and offending and into networks supportive of recovery. “Social cure” (Jetten et al., 2012), in which stronger social support (as measured in the question about the number of social network members one can talk to about important things) is clearly associated with more strengths and less barriers in recovery and greater increases in strengths while in recovery and greater reductions in recovery barriers. However, who is in your social network also matters as shown in our analyses. The higher the proportion of people in recovery in one’s social network and the lower the proportion of drug users in the network, the greater the total number of current strengths and the smaller the number of current barriers, which is consistent with the notion of “recovery contagion” (Best, 2019). We observed one interesting exception to this trend, as it appears that it is not beneficial to have a social network consisting exclusively of people in recovery, which is consistent with the “social cure” concept of the beneficial effects of belonging to multiple groups (Jetten et al., 2012).

The multi-variate analysis indicated positive associations for meaningful activities—with all of employment, education and volunteering associated with greater strengths in recovery. This is entirely consistent with previous quantitative (Best et al., 2011; Cano et al., 2017) and qualitative studies (De Maeyer et al., 2011; Pickering et al., 2020), showing the added value of meaningful activities. It further contributes to the evidence presented by the CHIME model of mental health recovery (Leamy et al., 2011; Best, 2019; Aga et al., 2021), indicating that positive social Connections generate Hope that in turn creates the conditions for Identity change that results from engaging in Meaningful activities which in turn enhances Empowerment.

There are some limitations to this study that merit mention. The sample is entirely self-selected—neither their recovery status nor their previous using experiences were examined or validated in any way. This also means that we cannot comment on the representativeness of the sample. As with all recovery studies, we have limited knowledge of the population and so commenting on the representativeness of the sample achieved is difficult, although it is worth noting that the size of the sample (in excess of 1,000) and the relatively balanced gender breakdown may suggest reasonable coverage. Nationality effects (as reported in the regression model) need to be treated with great caution as the recruitment strategy among the RUN members was much more limited (no fulltime researcher involved) than in the REC-PATH countries. Finally, the SABRS scale remains relatively untested and the accuracy of recollection of historical barriers and strengths and the potential for self-presentational bias in the current reporting of strengths and barriers cannot be validated or tested. We would suggest that future studies that use the Life in Recovery method consider reliability testing by repeated administration of the scale to at least examine test-retest consistency. Further, future research could administer the “in active addiction” component to those currently using substances to generate norms that could in principle validate the scores at a group level. Similarly, prospective designs could be used in future studies to assess both reliability and validity of both the LiR method and the resulting SABRS scores.

Nonetheless, the paper presents evidence on associations that are not reliant on sample representativeness and which suggests the importance of both familial and friendship effects in shaping recovery barriers and strengths. The SABRS scale is easy to administer and quick to complete and provides a measure of change that is not present in instruments that examine only current or past behaviours and do not offer the contrast offered by the LiR survey. Considerably more research is required to test the effects reported here prospectively, but what this paper indicates is both a “social cure” and further support for the importance of network transitions and domestic stability in building the recovery capital that is required to sustain recovery and wellbeing over time.

## Data Availability Statement

The raw data supporting the conclusions of this article will be made available by the authors, without undue reservation.

## Ethics Statement

The studies involving human participants were reviewed and approved by the Ghent University, Ethics Committee of the Faculty of Psychology and Educational Sciences, Belgium; the Sheffield Hallam University Ethics Committee, United Kingdom; and METC Erasmus MC, Netherlands. The patients/participants provided their written informed consent to participate in this study.

## Author Contributions

DB, AS, and LB undertook the data analysis and drafted a first version of the manuscript. MN and TM oversaw the data collection process and reviewed the draft versions. GN, DM, and WV wrote the introduction and discussion section and revised draft versions of the manuscript. All authors contributed to the conceptualisation, the drafting and reviewing of the manuscript, and revised draft version of this manuscript.

## Conflict of Interest

The authors declare that the research was conducted in the absence of any commercial or financial relationships that could be construed as a potential conflict of interest.

## Footnotes

1 https://www.rec-path.co.uk/
